# Epidemiology and etiology of brain cancer in Africa: A systematic review

**DOI:** 10.1002/brb3.3112

**Published:** 2023-06-14

**Authors:** Olivier Uwishema, Kristian Steen Frederiksen, Rawa Badri, Aishwarya Umesh Pradhan, Sanobar Shariff, Irem Adanur, Burhan Dost, Ignatius Esene, Gail Rosseau

**Affiliations:** ^1^ Department of Research and Education Oli Health Magazine Organization Kigali Rwanda; ^2^ Department of Research and Project Clinton Global Initiative University New York New York USA; ^3^ Faculty of Medicine Karadeniz Technical University Trabzon Turkey; ^4^ Danish Dementia Research Centre Rigshospitalet Copenhagen Denmark; ^5^ Mycetoma Research Centre Khartoum Sudan; ^6^ Faculty of Medicine University of Khartoum Khartoum Sudan; ^7^ Faculty of medicine Yerevan State Medical University Yerevan Armenia; ^8^ Department of Anaesthesiology and Reanimation Ondokuz Mayis University Faculty of Medicine Samsun Turkey; ^9^ Neurosurgery Division, Faculty of Health Sciences University of Bamenda Bambili Cameroon; ^10^ Department of Neurosurgery George Washington University School of Medicine and Health Sciences Washington, D.C. USA

**Keywords:** Africa, brain cancer, brain tumor, burden of disease, cancer

## Abstract

**Background:**

Cancer is a significant threat to public health and a leading cause of morbidity across the globe. Of all cancers, brain cancer can be particularly catastrophic as treatment often fails to achieve the desired degree of effectiveness and diagnosis remains associated with a high mortality rate. Africa, as a continent with resource‐limited countries, needs to allocate the necessary proper healthcare infrastructure to significantly reduce cancer rates and improve patient survival. In addition, the relative paucity of data within this field in Africa makes effective management a challenge.

**Objective:**

This review is aimed at elucidating the currently available evidence base with regard to the epidemiology and etiology of brain cancer within resource‐limited African countries. This review hopes to bring to the attention of the wider clinical community the growing burden of brain cancer within Africa and to encourage future research into this field of research.

**Methods:**

The available literature for this Systematic Review was searched on two bibliographic databases, PubMed and Scopus, using an individually verified, prespecified approach. In addition, the Global Cancer Observatory and Global Burden of Disease databases were also utilized. Studies reporting on the epidemiology, etiology, and impact of brain cancer in Africa were suitable for inclusion. The level of evidence of the included studies was considered as per the Centre for Evidence‐Based Medicine recommendations.

**Results:**

Out of the four databases searched, 3848 articles were initially screened rigorously, filtered into 54 articles, and finally assessed qualitatively and quantitatively. We have demonstrated a poor survival rate and lack of proper funds/resources necessary to report, identify, and treat cases, as well as the dearth of comprehensive research on the subject of brain cancer that has become a challenging healthcare concern in many African developing nations. Also, because of the gradual improvement in healthcare facilities and the increasing population within many countries in Africa, the number of patients with central nervous system and intracranial tumors is rising specifically in the elder population. In addition, the population in West Africa is at a higher risk of HIV‐related malignancies due to the high prevalence of HIV in West Africa. The burden of brain cancer in Africa is increasing in comparison with the developed parts of the world in which it is decreasing. Moreover, the mismanagement of cancers in Africa leads to higher morbidity and mortality and decreased quality of life.

**Conclusion:**

This study addresses the burden of brain cancer as a major public health crisis in Africa. Improved treatment modalities and access to screening are required to better address the burden of this disease. Therefore, there is a clear need for more substantial and comprehensive research on etiology, epidemiology, and treatment of brain cancer within Africa to understand its epidemiological distribution and provide a means for managing and reducing the associated morbidity and mortality.

## 1 INTRODUCTION

Primary central nervous system (CNS) cancers are malignant tumors that grow in the different anatomical regions of the CNS. However, more than 90% occur in the brain (Cancer.net, 2018). Considering that primary CNS cancers account for only 1.3% of all cancers (NCI, 2017), this form of malignancy is generally considered uncommon in adults.

Despite past efforts, only three risk factors have been evidenced to be directly linked to brain cancer: radiation, family history, and a weakened immune system (Recht, 2019). However, only 5% of patients with brain tumors have a family history of a malignant disease (Savage, 2018).

Cancer has previously been considered to affect mainly high‐income countries, yet several studies show that resource‐limited countries have an increasing burden of oncological diseases (Ferlay et al., 2010; Kanavos, 2006). One possible explanation for this is that these countries have witnessed a growth in their ageing population within the last 20 years (World Bank, 2021). Changes in demographic structure have set in motion a transition from communicable diseases that affect predominantly younger people toward noncommunicable disorders like cancer that more commonly affect adults (Bollyky, 2020). This has meant that developing countries in Africa now have to manage the double burden of both communicable and noncommunicable diseases (Pradhan et al., 2022; Uwishema, Ayoub, et al., 2022; Uwishema, Berjaoui, et al., 2022; Uwishema, Frederiksen, et al., 2022; Uwishema, Mahmoud, et al., 2022; Uwishema, Onyeaka, et al., 2022).

Cancer mortality in sub‐Saharan Africa (SSA) has increased by 45% since 2000, resulting in more than 500,000 deaths each year (Bollyky, 2020). In contrast, cancer death rates have mostly decreased within developed countries. In addition, relative to other malignancies, brain cancer has a low survival rate with 5‐year survival being reported at only 32% and median survival being less than 2 years (NCI, 2017; Ostrom et al., 2013). Many patients who survive will also suffer from residual disability (Lapointe et al., 2018).

The histologic profile and method of treatment are two important prognostic factors (Lapointe et al., 2018; Savage, 2018). Nevertheless, the diagnosis and treatment of brain cancer require highly specialized healthcare infrastructure including facilities and medical personnel that are beyond the health budgets of many African countries (WHO | Regional Office for Africa, 2018; Wilson et al., 2018). Only 35% of developing nations had pathology facilities in public hospitals and institutions in 2015, according to the World Health Organization (WHO) (WHO | Regional Office for Africa, 2018). It is therefore evident that developed countries have far greater cancer research and treatment facilities than lower income countries.

The inadequate health resources of many African countries have led to the underestimation of brain cancer incidence (Ngulde et al., 2015). This results in underreporting of the negative impacts that brain cancer has on society (Ngulde et al., 2015). The lack of a healthcare system able to provide adequate diagnostic services and treatment poses a severe challenge to resource‐limited countries. To reduce cancer rates and improve survival, multimodal approaches and support are required. Increased personalized medicine, greater screening access, and different treatment modalities are essential to address the burden of cancer, specifically CNS tumors (Bollyky, 2020).

To quote a Kalenjin Kenyan proverb: “We should put out the fire while it is still small.” To help remedy this inadequate information regarding brain cancer in Africa, a basic comprehension of this field is needed first by providing a brief account of different factors such as prevalence, risk factors, and diagnosis. However, it is of utmost importance to obtain a superior understanding of brain cancers’ effects within Africa.

Therefore, to assess the existing data on brain cancer in Africa, as well as to collate and consolidate the current evidence on its epidemiology and etiology, this literature review attempts to provide a clearer evaluation of the disease's burden, which will inform and aid countries in addressing this issue.

## 2 METHODOLOGY

### 2.1 Data source

The data for this scoping review were gathered between March 20 and May 20, 2021. Google Scholar, PubMed, Scopus, OVID Medline, and Ebscohost as well as the WHO Global Cancer Observatory (GCO) and Global Burden of Disease (GBD) were the databases screened for literature using an independently approved, predetermined search strategy (Patel et al., 2019; WHO, 2020). Africa, brain cancer, brain tumor epidemiology, and disease burden were the terms used in the search.

### 2.2 Study selection

Two authors were in charge of implementing the inclusion and exclusion criteria for study selection (O.U. and K.S.F.). The following criteria were specified as inclusion criteria: (i) publications discussing epidemiology; (ii) articles discussing etiology; (iii) the effect of brain cancer in Africa; (iv) studies published after 2005; and (v) articles published in English. Commentaries, editorials, and nonclinical investigations were excluded.

### 2.3 Study population

All African continent populations were included in the study; however, there were no data on the North African population.

### 2.4 Study screening

The Centre for Evidence‐Based Medicine criteria (Howick et al., 2011; OCEBM Levels of Evidence Working Group, 2011) were used to assess the level of evidence in the included studies.

### 2.5 Bias assessment

The inclusion and exclusion criteria for study selection were formulated by two authors (O.U. and K.S.F.) in blind duplicate. Additionally, the risk of bias was independently inspected and rated at the study and outcome levers in accordance with the Cochrane Handbook for Evaluating Study Quality (Higgins et al., 2008).

### 2.6 Difference of opinion

A difference of opinion was settled via dialogue and consultation with a third author (I.E.).

## 3 RESULTS

**FIGURE 1:**
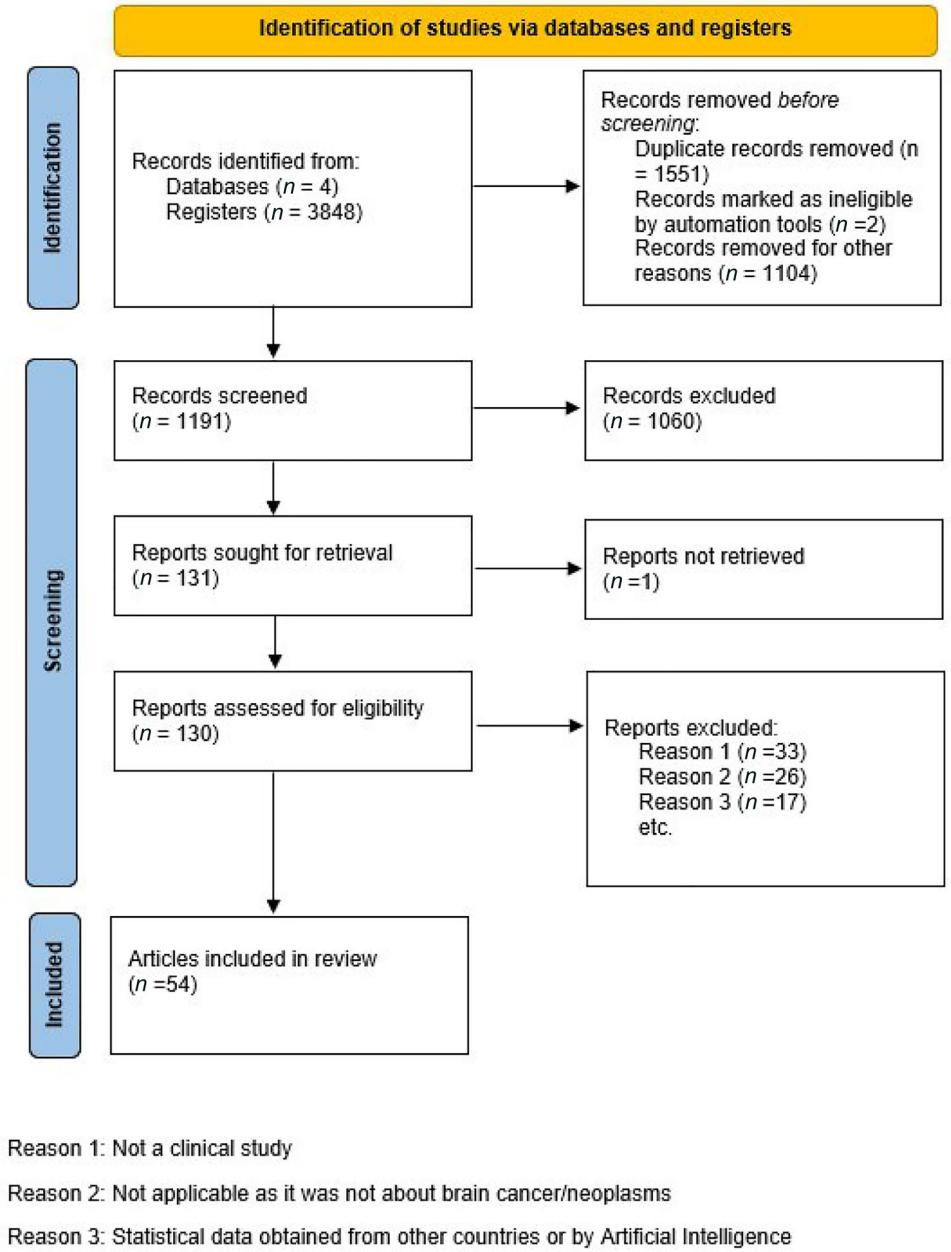
The results of our comprehensive data search spanning over four databases described virtually by the Prisma flow chart.

Four databases were searched (National Library of Medicine, Scopus, OVID Medline, and Ebscohost). The database search returned 3848 articles. After deduplication and title and abstract screening, 1191 articles (30.95 %) remained. Most articles were excluded because they aggregated cancer of all organs without distinctions (*n* = 1060), while another one was excluded because of lack of retrieval. Only 130 articles were included after the full‐text screening and further 77 articles were removed from the final cluster of articles. The reasons for exclusion ranged from irrelevant subjects to not focusing on brain tumor/neoplasms and statistical data pertaining to countries not from Africa or artificial intelligence predictive models. Fifty‐three studies were finally included for quantitative and qualitative analysis (Figure 1).

### 3.1 Epidemiology

Epidemiological data on brain cancers are lacking in Africa; thus, estimating prevalence and incidence is difficult. Of the total African population, only 11% is covered by cancer registries. Furthermore, cancer registries that meet the Cancer Incidence in Five Continents (CI5) inclusion criteria cover just 1% of the covered population (Ferlay et al., 2010). However, due to the WHO GCO program, international studies, and local hospital research, the prevalence and epidemiology of brain cancer within Africa can now be more accurately estimated.

**FIGURE 2:**
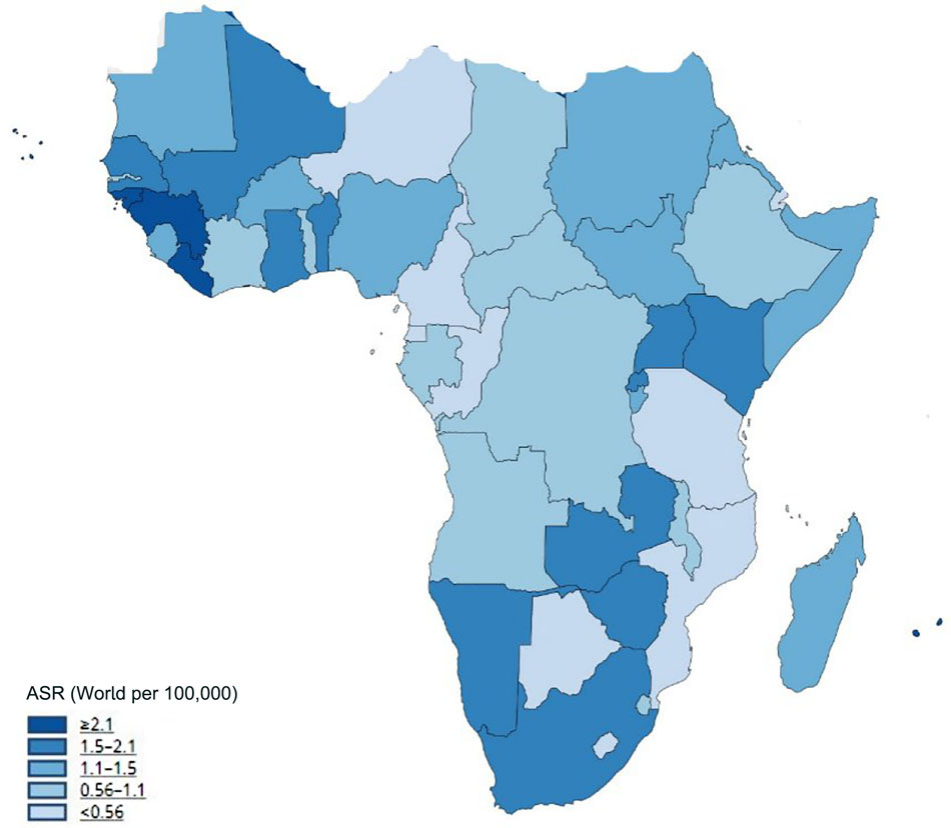
Age‐standardized rate incidence of brain cancer in Africa per 100,000 individuals. *Source*: GLOBOCAN 2020. Graph production: International Agency for Research on Cancer (IARC) 2021 (http://gco.iarc.fr/today) and World Health Organization (WHO). Copyright authorization: The International Agency for Research on Cancer/World Health reproduced with permission of IARC/WHO.

**FIGURE 3:**
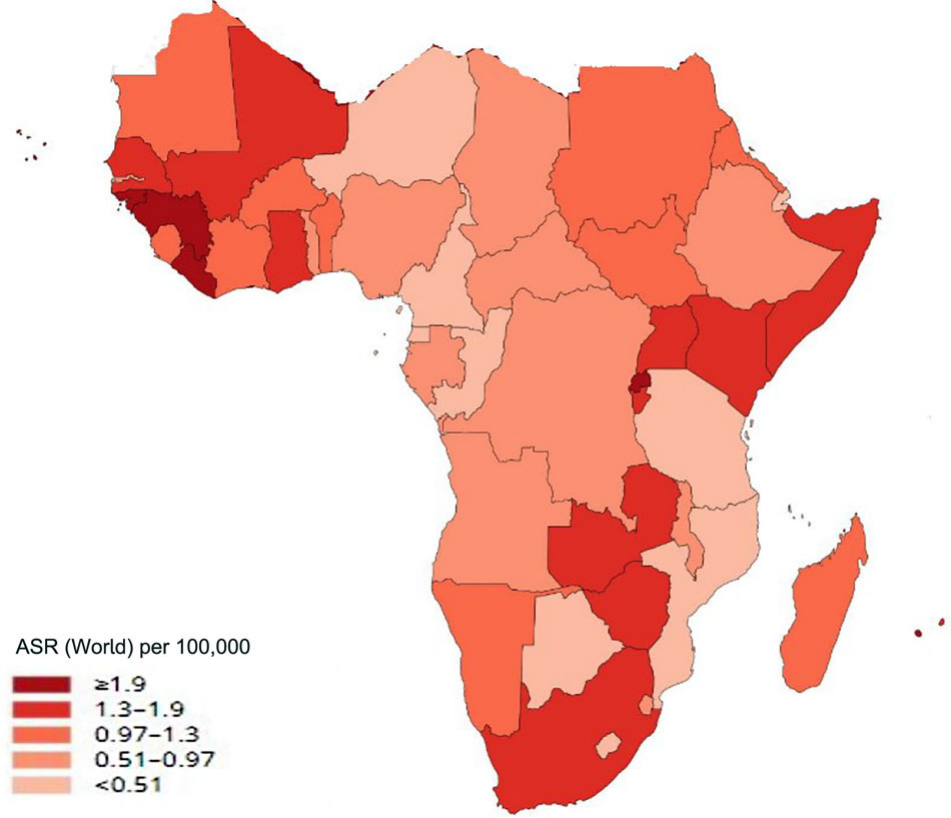
Age‐standardized rate mortality of brain cancer in Africa per 100,000 individuals. *Source*: GLOBOCAN 2020. Graph production: International Agency for Research on Cancer (IARC) 2021 (http://gco.iarc.fr/today) and World Health Organization (WHO). Copyright authorization: The International Agency for Research on Cancer/World Health reproduced with permission of IARC/WHO.

Based on GCO, in 2020, approximately 9169 novel cases of brain cancer were estimated by SSA, accounting for 3.0% of the overall cases across the globe (WHO, 2020), while around 14.1% of the world population live in the continent (World Bank, 2021). However, in 2016, a Lancet study (Patel et al., 2019), based on GBD estimates, reported 12,754 cases of brain cancers (3.9% of the global total) in this region. This same study reported a 13.9% change in brain cancer incidence at age‐standardized rates (ASRs) between 1990 and 2016 in these countries (Patel et al., 2019). In addition, the African 5‐year prevalence was 4.9% in 2020 (WHO, 2020) (Figures 2 and 3).

**FIGURE 4:**
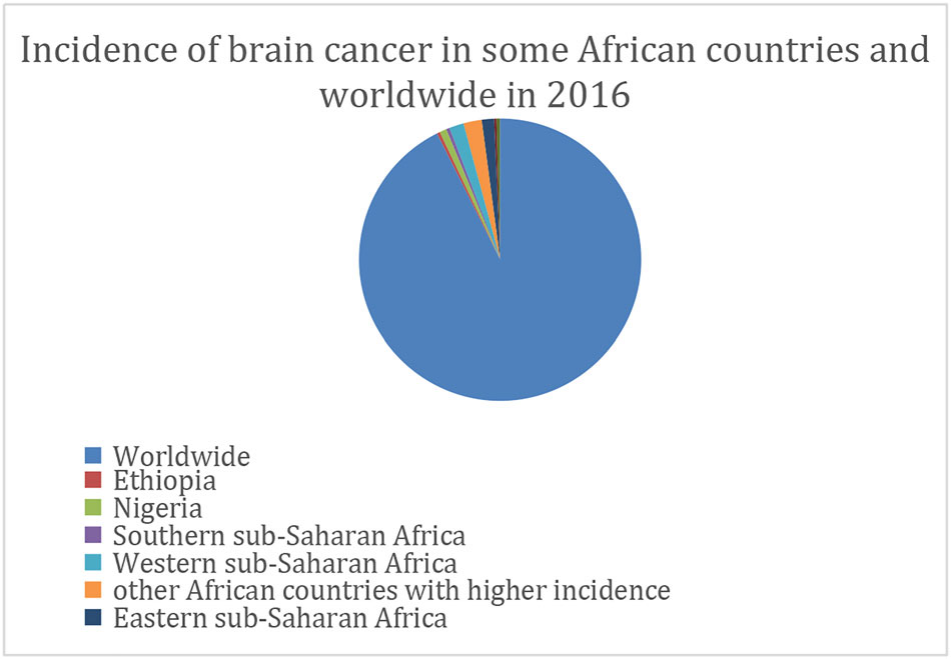
This figure describes the incidence of brain cancer in African countries and worldwide in 2016 (Patel et al., 2019).

Minimal research has been undertaken on the incidence of brain tumors in Africa at a national level. According to a Nigerian study published in 2011, brain cancer accounted for 3.6% of cancer cases in Lagos and Ibadan, coming sixth after breast, cervical, fibroids, liver, and stomach cancer (Awodele et al., 2011), while the global rate is 1.3% (NCI, 2017). More recently in 2020, the reported incidence of brain tumors in Nigeria was 1798 (WHO, 2020), which means that Nigeria reported over 50% new cases of the average 5‐year prevalence (3568) in a single year (WHO, 2020). Moreover, brain cancer was reported as the 13th most common cancer in Nigeria that year, whereas it was only 19th in the world (WHO, 2020). These data discrepancies raise concerns about the credibility of brain cancer reports in Nigeria. This may reflect that primary brain tumors are either overreported due to misdiagnosing brain metastasis as primary cancer since many African countries lack basic pathology services (WHO | Regional Office for Africa, 2018) or there is a worrying increase in incidence.

A few other studies have reported country‐specific epidemiological data. Within 11 years in Abidjan, Republic of Côte d'Ivoire, 362 brain cancer cases were registered from just one hospital, and 15.74% (*n* = 57) of these cases were present in children (Broalet et al., 2008), which is in concordance with the international data (Merchant et al., 2010). Furthermore, 30 brain tumors were seen in a single hospital in Ghana over 2 years, while tumors were shown to be the source of 3.1% of hydrocephalus cases in The Republic of Niger (Morhason‐Bello et al., 2013). Although these numbers are consistent with internationally reported data, they lack reliability as they are based on local hospital registries.

The maps show the different incidence and mortality ASRs in the SSA‐hub in Figures 2 and 3.

### 3.2 Risk factors

Exposure to radiation, genetic factors, age, and a compromised immune system are a few of the many risk factors implicated in the development of brain cancer (Recht, 2019). Owing to the increasing population and gradually improving medical facilities within many African countries, there has been a rise in the number of patients of all ages, specifically in the geriatric patient population. This can be partially attributed to the developments in antiretroviral therapies that have prolonged the expected life spans for HIV‐infected patients (The African Regional Health Report 2017; WHO | Regional Office for Africa, 2018). However, almost half of all HIV/AIDS patients now go on to develop associated malignancy (Mwamba et al., 2012; Sasco et al., 2010).

As per cancer registries, the incidence of CNS and intracranial tumors increases with age, with elderly people having the highest rates (Cancer Research UK, 2015). When this is considered alongside the fact that African countries have seen an approximate 14% brain cancer increase in ASR between 1990 and 2016 (Patel et al., 2019) and that according to World Bank data the SSA population's life expectancy at birth has witnessed an increase since the 1990s, from 50.21 years in 1990 to 61.27 years in 2018 (World Bank, 2021), it seems likely that the increase in brain cancer rates in Africa can be in part attributed to its ageing population. Moreover, brain malignancies are also the second most common type of malignancies in pediatric patients (Cancer.net, 2012) that has increased almost twofold in Africa in the last two decades and children mortality decreased by 58% (UNICEF DATA, 2019). Paternal preconceptional workers exposed to aromatic polycyclic hydrocarbons was linked to elevated risks of all juvenile brain cancers and astroglial tumors, according to large worldwide population‐based case–control studies. Consequently, increases in brain cancer incidence can be affected by demographic change (Idowu & Idowu, 2008)

The main acquired cause of glial and meningeal neoplasms is ionizing radiation (Idowu & Idowu, 2008; Savage, 2018). This is supported by evidence that shows that exposure to radiation increases the risk by three‐ to 10‐fold with a latency period of 10–20 years postexposure (Idowu & Idowu, 2008). This is particularly relevant within Africa for two reasons. The first is that the relatively recent medical developments in the continent, which were previously not available, are leading to greater use of radiation for treatment and diagnosis (IAEA, 2016). The second is that the International Atomic Energy Agency (IAEA) reports that, in most African countries, there is a critical shortage of qualified medical physicists in healthcare facilities that results in patients receiving nonoptimized radiation, resulting in exposure to the harmful effects of radiation (IAEA, 2016). Therefore, decreasing radiation exposure represents a possible modifiable risk factor for CNS malignancies within Africa.

Furthermore, several oncogenic viruses, namely, HIV, are highly prevalent in West Africa, putting the population at a higher risk of HIV‐related malignancies (Kelly & Lekgwara, 2020). Non‐Hodgkin lymphoma (NHL) affects 10% of HIV patients, of which primary CNS lymphoma (PCL) is a common form (Cheng et al., 2020). NHL is said to occur due to the immunocompromised state of the body because of HIV and other viruses such as Epstein–Barr virus (Cheng et al., 2020; Kelly & Lekgwara, 2020). Patients diagnosed with AIDS have shown a far greater incidence of PCL (Grogg et al., 2006; O'neill et al., 2015). Despite discoveries and incredible breakthroughs in antiretroviral medication, the risk of neurological consequences such as primary CNS lymphoma remains high and adversely impacts not only the physical health but also the psychological well‐being of the patients (Uwishema et al., 2022). Therefore, the study of CNS malignancies in Africa is closely associated with statistics of infection and communicable diseases, and to reduce the incidence of cancer in Africa, reduction of HIV infection rates is imperative.

Finally, some studies suggest that eating a nutritious diet rich in fruit, greens, and vitamins C and E may prevent the development of brain tumors, but the low nutritional status in certain areas of Africa may act as an additional etiological factor (Bielecka & Markiewicz‐Żukowska, 2020; Kimokoti & Hamer, 2008).

### 3.3 Burden

Ranked as the second major cause of mortality worldwide, cancer accounted for an estimated 9.6 million deaths in 2018 (National Cancer Institute, 2020). While communicable diseases create an overwhelming health burden in Africa, other diseases, mainly cancer, are underemphasized (Parkin et al., 2008), since in 2020, the WHO classified many of the SSA countries as those with the greatest cancer premature mortality (Sung et al., 2021). In resource‐limited countries, brain neoplasms are often overlooked and research on it is limited, which may be due to difficulty in their accurate diagnosis. However, brain and other CNS malignancies are a serious cause of mortality and morbidity, and their diagnosis and treatment need considerable issuance of resources as well as highly specialized diagnostic and therapeutic tools (Patel et al., 2019).

According to WHO reports in 2015, treatment services are three times greater in developed countries compared to in low‐income countries (WHO | Regional Office for Africa, 2018). Consequently, specialized cancer care is not adequately accessible in resource‐limited regions of the world. Additionally, only one in five developing countries has the required data to manage cancer policies (WHO | Regional Office for Africa, 2018). According to a 2009–2010 Hospital Based Cancer Registry (HBCR) report that contained data from 11 cancer registries in Nigeria, 50% of male and 33% of female cases of the 6484 recorded cases were not documented by type, and CNS tumors were completely missed and unaccounted for (Akinde et al., 2015).

Furthermore, worldwide estimates exhibit a significant variance in the incidence of CNS malignancies, with the standardization of age in different countries varying between 0.01 and 12.7 in males and 0.01 and 10.7 in females (Kalan Farmanfarma et al., 2019). The African continent has the lowest incidence, while the highest level is in northern Europe (Kalan Farmanfarma et al., 2019). However, it is suggested that this is likely due to severe underreporting in Africa as a direct result of inadequate diagnostic and reporting facilities.

Similar trends in mortality rates are observed across gender and economic development (Bondy et al., 2008; Ferlay et al., 2010), and the mortality rates reported are implausibly low within African data, again suggesting inaccurate or inadequate data reporting. In fact, in 2015, the share of brain cancer mortality rate was around 3% worldwide but only 1.75% in the SSA (Roser & Ritchie, 2018). For example, at Lagos University Teaching Hospital, Nigeria, 4.7% of all deaths were due to cancer, with CNS‐associated malignancies responsible for 4.9% of this death rate (Jedy‐Agba et al., 2012). In contrast to population‐based registries, which cover just 7.2% of the overall population, these data are taken from HBCR (Akinwande et al., 2009).

Additionally, the growth in ASR of disability‐adjusted life‐years (DALYs) is positive (22.5%) in the low‐sociodemographic index (SDI) and negative (−13.4%) in medium‐SDI countries (Higgins et al., 2008). These changes show that the burden of brain cancer is increasing in African countries, while it is decreasing in more developed parts of the world. These DALYs reported correspond to brain cancer solely. Moreover, even though primary CNS cancer comes 12th in terms of incidence worldwide, its burden is eighth (Roser & Ritchie, 2018).

Yet, after accounting for the increase in CNS malignancies between 1990 and 2016, as well as its role in causing significant morbidity and mortality worldwide, the burden of CNS cancer in Africa is underreported. Variability in diagnostic and reporting techniques, as well as unidentified environmental and genetic risk factors, is likely to contribute to a considerable geographical and regional disparity in the incidence of CNS malignancies.

Consequently, there is a mismanagement of cancers in Africa, leading to higher morbidity and mortality and a worse quality of life. Despite its limited resources and diagnostic abilities, Africa has a huge battle to fight the dual burden of the rising communicable diseases and the ever‐present noncommunicable diseases.

This review reveals the scarcity of proper reporting on the disease and suggests that the real burden of brain cancer, already positive, is yet to be appropriately reported in Africa.

The table below (Table 1) summarizes the deaths, incidence, and DALY in ASR, according to GBD data (Patel et al., 2019).

**TABLE 1:** Deaths, incident cases, and DALYs for CNS cancer in 2016 and percentage change between 1990 and 2016 in age‐standardized rates by location.

	Deaths (95% UI)		Incidence (95% UI)		DALYs (95% UI)	
	2016 counts	Percentage change in age‐standardized rates between 1990 and 2016	2016 counts	Percentage change in age‐standardized rates between 1990 and 2016	2016 counts	Percentage change in age‐standardized rates between 1990 and 2016
Global	227,039 (204,784 to 241,279)	−2.2 (−7.7 to 8.0)	329,673 (298,926 to 348,845)	17.3 (11.4 to 26.9)	7,659,974 (6,922,776 to 8,280,367)	−10.0 (−16.4 to 2.6)
Angola	276 (205 to 364)	33.6 (0.5 to 85.1)	296 (253 to 349)	2.9 (−8.7 to 13.8)	14,106 (10,646 to 18,765)	32.4 (1.5 to 77.3)
Benin	158 (124 to 183)	30.1 (−1.3 to 77.7)	146 (115 to 165)	25.6 (0.4 to 56.0)	7028 (5377 to 8265)	31.1 (−2.1 to 66.0)
Botswana	30 (15 to 49)	30.8 (−38.2 to 179.2)	32 (26 to 41)	28.1 (−0.7 to 126.6)	196 (620 to 1856)	26.9 (−37.7 to 173.7)
Burkina Faso	237 (180 to 276)	27.0 (−6.6 to 71.2)	222 (170 to 260)	25.6 (−0.4 to 61.5)	11,393 (8399 to 13,764)	34.6 (−2.8 to 76.9)
Burundi	125 (100 to 152)	11.3 (−11.4 to 39.3)	129 (111 to 148)	1.0 (−13.4 to 18.8)	5816 (4543 to 7226)	11.4 (−13.5 to 42.3)
Cameroon	432 (264 to 699)	33.9 (−4.3 to 82.1)	385 (248 to 607)	28.3 (0.4 to 59.0)	19,900 (11,947 to 32,581)	40.8 (−0.3 to 84.2)
Cape Verde	11 (9 to 16)	31.2 (−8.1 to 139.7)	11 (10 to 16)	19.3 (−10.0 to 111.4)	487 (407 to 598)	26.6 (−6.0 to 121.5)
Central African Republic	54 (42 to 66)	11.2 (−12.7 to 42.7)	67 (57 to 77)	6.8 (−2.9 to 21.2)	2467 (1889 to 3128)	13.0 (−12.9 to 43.1)
Central sub‐Saharan Africa	1206 (945 to 1 428)	15.5 (2.7 to 29.8)	1384 (1177 to 1578)	0.3 (−13.6 to 10.7)	58,205 (48,384 to 69,826)	13.9 (−3.8 to 30.3)
Chad	162 (125 to 193)	23.0 (−3.0 to 69.1)	162 (125 to 188)	22.5 (0.3 to 54.8)	8040 (6101 to 9625)	29.7 (−0.3 to 66.5)
Comoros	11 (9 to 15)	24.5 (−4.9 to 83.3)	10 (9 to 12)	17.4 (−1.3 to 59.2)	496 (400 to 644)	20.7 (−6.5 to 73.7)
Congo (Brazzaville)	71 (48 to 120)	18.7 (−12.7 to 60.8)	75 (54 to 126)	7.6 (−6.5 to 21.6)	3232 (2096 to 5695)	20.1 (−10.8 to 60.4)
Côte d'Ivoire	264 (214 to 316)	27.7 (−2.6 to 92.9)	221 (186 to 249)	16.6 (−6.3 to 66.2)	11,271 (9314 to 13,242)	28.1 (−0.5 to 89.2)
Djibouti	14 (11 to 18)	34.2 (−11.7 to 188.5)	13 (10 to 14)	25.8 (−1.0 to 141.0)	593 (441 to 760)	27.9 (−12.5 to 164.0)
DR Congo	762 (532 to 953)	10.2 (−9.0 to 28.6)	901 (706 to 1 043)	−2.1 (−22.0 to 14.1)	36,573 (28,328 to 45,197)	7.7 (−17.2 to 29.4)
Eastern sub‐Saharan Africa	4868 (4299 to 5911)	27.6 (5.5 to 72.3)	4610 (4143 to 5265)	14.2 (−0.3 to 47.9)	217,746 (192,461 to 253,543)	23.5 (6.2 to 58.7)
Equatorial Guinea	12 (6 to 22)	22.2 (−32.1 to 105.0)	15 (11 to 26)	17.5 (−5.6 to 59.0)	495 (268 to 930)	12.9 (−34.0 to 76.3)
Eritrea	73 (59 to 97)	36.8 (−0.2 to 114.5)	66 (56 to 79)	22.7 (−1.0 to 71.6)	3265 (2695 to 4200)	34.4 (1.9 to 104.4)
Ethiopia	1305 (1059 to 1720)	23.4 (−2.6 to 58.5)	1164 (1032 to 1437)	5.3 (−5.7 to 21.2)	53,464 (43,380 to 69,382)	17.9 (−5.6 to 45.6)
Gabon	32 (21 to 55)	34.3 (−7.0 to 97.0)	30 (22 to 53)	19.1 (−1.3 to 55.2)	1331 (858 to 2412)	35.0 (−5.7 to 90.1)
The Gambia	18 (15 to 22)	17.6 (−7.0 to 54.6)	17 (14 to 20)	8.4 (−7.1 to 30.8)	827 (689 to 983)	14.5 (−8.7 to 44.3)
Ghana	652 (551 to 769)	24.2 (−2.1 to 74.1)	608 (522 to 676)	5.8 (−11.5 to 46.8)	29,701 (23,563 to 35,300)	20.7 (−3.0 to 65.4)
Guinea	193 (153 to 250)	21.5 (−8.6 to 68.8)	167 (142 to 201)	12.9 (−7.7 to 46.7)	7153 (5876 to 9138)	18.7 (−7.0 to 58.5)
Guinea‐Bissau	31 (23 to 38)	23.7 (−3.3 to 61.0)	26 (20 to 31)	20.1 (0.1 to 38.5)	1382 (1045 to 1657)	26.6 (−1.7 to 58.6)
Kenya	414 (287 to 537)	35.4 (5.5 to 153.7)	491 (285 to 615)	15.1 (−0.3 to 106.8)	18,590 (12,136 to 24,765)	34.7 (6.7 to 141.3)
Lesotho	25 (17 to 37)	38.7 (−18.6 to 242.6)	29 (22 to 39)	37.8 (−3.2 to 212.2)	1036 (749 to 1485)	40.3 (−17.3 to 250.7)
Liberia	56 (45 to 66)	28.9 (2.3 to 58.5)	57 (47 to 65)	18.5 (−1.4 to 36.3)	2409 (1914 to 2868)	27.7 (1.9 to 55.9)
Madagascar	289 (232 to 352)	22.0 (−10.3 to 80.6)	309 (270 to 347)	16.3 (−1.9 to 54.2)	12,852 (10,415 to 15,830)	19.8 (−10.3 to 71.1)
Malawi	167 (132 to 207)	17.8 (−16.7 to 87.6)	162 (146 to 184)	10.6 (−7.9 to 46.3)	7315 (5873 to 9087)	10.5 (−19.6 to 60.0)
Mali	154 (124 to 193)	7.8 (−14.7 to 35.3)	159 (142 to 185)	2.2 (−8.3 to 12.7)	6968 (5578 to 9098)	3.2 (−18.2 to 26.0)
Mauritania	64 (44 to 84)	21.2 (−11.5 to 59.4)	56 (44 to 66)	15.6 (−3.3 to 34.7)	2816 (2002 to 3586)	28.0 (−8.2 to 69.3)
Mozambique	561 (442 to 742)	12.2 (−14.1 to 58.0)	489 (411 to 632)	9.4 (−7.8 to 42.6)	28,852 (22,684 to 36,222)	8.4 (−12.6 to 42.1)
Namibia	23 (15 to 30)	16.5 (−29.1 to 101.0)	24 (19 to 27)	16.6 (−4.1 to 65.6)	951 (664 to 1231)	14.8 (−27.3 to 91.4)
Niger	206 (111 to 290)	18.3 (−11.0 to 54.0)	212 (116 to 276)	12.3 (−7.7 to 33.6)	9523 (5122 to 13,282)	18.2 (−13.9 to 59.4)
Nigeria	2165 (1542 to 3043)	22.9 (−4.2 to 53.2)	2632 (1938 to 3506)	15.8 (−0.1 to 33.5)	106,875 (73,141 to 1,56,444)	26.5 (−1.5 to 60.0)
Rwanda	153 (122 to 190)	35.6 (8.1 to 71.7)	143 (126 to 161)	6.5 (−3.5 to 19.3)	6941 (5543 to 8436)	32.7 (7.1 to 64.4)
São Tomé and Principe	2 (1 to 2)	22.2 (−11.4 to 75.9)	2 (1 to 2)	16.1 (−6.2 to 51.4)	78 (59 to 100)	16.0 (−11.7 to 56.7)
Senegal	231 (186 to 267)	36.8 (8.6 to 74.2)	190 (152 to 216)	20.8 (−1.2 to 40.9)	10,460 (8278 to 12,340)	39.7 (10.8 to 66.9)
Sierra Leone	88 (68 to 116)	33.6 (−4.5 to 89.5)	91 (69 to 117)	29.4 (−0.1 to 67.8)	4115 (3041 to 5544)	34.1 (−4.4 to 78.8)
Somalia	125 (100 to 153)	16.2 (−6.8 to 49.9)	123 (103 to 137)	11.7 (−0.6 to 34.6)	5564 (4375 to 6798)	13.9 (−7.5 to 43.9)
South Africa	822 (687 to 915)	27.9 (−0.4 to 83.5)	967 (804 to 1 049)	25.9 (2.4 to 75.7)	34,233 (26,639 to 39,366)	35.9 (−0.6 to 88.2)
South Sudan	121 (65 to 174)	26.1 (−6.1 to 94.3)	139 (80 to 181)	11.6 (−0.8 to 52.4)	5346 (2882 to 7503)	22.0 (−6.9 to 75.8)
Southern sub‐Saharan Africa	1177 (982 to 1317)	27.6 (4.5 to 94.6)	1292 (1083 to 1388)	17.7 (1.5 to 74.1)	50,339 (40,967 to 55,989)	31.0 (5.9 to 91.1)
Swaziland	14 (9 to 20)	14.4 (−27.3 to 108.1)	16 (12 to 20)	16.3 (−2.7 to 81.8)	623 (414 to 882)	18.3 (−22.2 to 113.3)
Tanzania	775 (636 to 1 015)	32.0 (−5.1 to 112.5)	662 (587 to 797)	22.0 (−1.1 to 82.1)	34,825 (28,826 to 44,349)	28.4 (−3.3 to 96.1)
Togo	114 (83 to 147)	36.0 (−2.1 to 100.1)	102 (76 to 127)	30.7 (−0.5 to 75.8)	5063 (3685 to 6422)	37.6 (−3.0 to 87.4)
Uganda	470 (361 to 588)	45.0 (−1.0 to 121.9)	473 (379 to 553)	29.8 (−1.7 to 81.0)	21,876 (17,588 to 26,779)	42.3 (3.9 to 112.3)
Western sub‐Saharan Africa	5238 (4210 to 6459)	25.4 (4.8 to 53.6)	5468 (4268 to 6635)	16.3 (0.5 to 32.6)	245,490 (189,605 to 3,10,117)	27.3 (5.2 to 47.3)
Zambia	264 (170 to 454)	58.7 (−8.2 to 191.6)	233 (169 to 371)	39.7 (−0.8 to 141.0)	11,948 (8066 to 19,533)	48.5 (−8.3 to 160.5)
Zimbabwe	263 (206 to 333)	39.2 (1.2 to 154.5)	224 (192 to 256)	−1.9 (−17.5 to 72.9)	12,300 (9744 to 15,435)	34.4 (−1.2 to 137.5)

*Note*: Every rate is given in terms of 100,000 person‐years. The 95% uncertainty intervals (UIs) for each estimate, which were obtained from the 2.5th and 0.775th percentiles of 1000 draws, were generated for all estimations between 1990 and 2016. Source: Global Burden of Diseases, Injuries, and Risk Factors Study 2016 (Patel et al., 2019) (Figure 4). Copyright authorization: The table is reproduced with permission.

Abbreviations: CNS, central nervous system; DALYs, disability‐adjusted life‐years; UI, uncertainty interval.

## 4 CONCLUSION

In conclusion, this study addresses the major public health crisis caused by brain cancer in the African continent, which has been overshadowed by the historic focus on communicable infectious diseases. First, the study revealed unreliable and discrepant data; thus, it would be inaccurate to report that the incidence increase is true across all African countries. However, this review recognizes several contributing factors to the increasing prevalence of brain tumors within Africa and identifies these factors as individual opportunities for strategies to address the growing burden of this disease. If brain tumors are to be effectively managed and controlled within Africa, then these factors must be addressed in a multimodal and multidisciplinary manner.

Consequently, the lack of a proper and well‐structured healthcare system in the African continent has led to an incomplete understanding of brain cancer's actual impacts: diagnosis, symptoms, risk factors, screening, and treatment of brain cancer are incompletely studied. However, knowledge and awareness about these deficiencies are crucial in providing appropriate medical services and establishing a trustworthy database of brain cancer statistics in Africa. This should be a priority, as the incidence of brain cancer is increasing due to demographic changes and other modifiable factors such as radiation, HIV, and nutrition.

Secondary prevention of brain cancer and the establishment of effective strategies are needed to overcome the population's lack of awareness of this rising threat. Further efforts should focus on fighting the increasing incidence, morbidity, and mortality of these cancers as well as their impacts on the community. This requires a strong healthcare system that consists of well‐structured healthcare facilities and the incorporation of public health agencies and brain cancer societies. It will also need international organizations to collaborate and fund research regarding epidemiology, etiology, and treatment of brain cancer in Africa, as well as to share their experience.

This review recommends that evidence‐based brain cancer treatment and prevention should be implemented in Africa, and a greater degree of related research should be done locally to bridge the existing knowledge gap. These requirements, which are currently considered obstacles toward controlling brain cancer in Africa, can be overcome by ensuring adequate human resources and adequate budgetary allocation to the health sector and brain cancer research within Africa.

## AUTHOR CONTRIBUTIONS

Olivier Uwishema conceptualized the idea of the study, administered the project, and reviewed and designed the manuscript. Kristian Steen Frederiksen performed supervision, reviewed the final draft, edited the first and second drafts, and made critical comments. Olivier Uwishema reviewed and edited the first draft. Burhan Dost and Ignatius Esene reviewed and edited the third draft. Gail Rosseau reviewed and edited the fourth draft. Rawa Badri reviewed and edited the final draft and performed data analysis and interpretation. All authors collected and assembled the data, wrote the manuscript, and given final approval to the manuscript.

## Funding information

This study has not received any financial support.

## CONFLICT OF INTEREST STATEMENT

The authors declare no conflicts of interest.

### PEER REVIEW

The peer review history for this article is available at https://publons.com/publon/10.1002/brb3.3112.

## Data Availability

The data used to validate the findings of the study are included within the article.
